# Visualizing T Cell Migration *in situ*

**DOI:** 10.3389/fimmu.2014.00363

**Published:** 2014-07-29

**Authors:** Alexandre P. Benechet, Manisha Menon, Kamal M. Khanna

**Affiliations:** ^1^Department of Immunology, University of Connecticut Health Center, Farmington, CT, USA

**Keywords:** CD8 T cells, imaging techniques, intravital microscopy, migration, T cells, infections

## Abstract

Mounting a protective immune response is critically dependent on the orchestrated movement of cells within lymphoid tissues. The structure of secondary lymphoid organs regulates immune responses by promoting optimal cell–cell and cell–extracellular matrix interactions. Naïve T cells are initially activated by antigen presenting cells in secondary lymphoid organs. Following priming, effector T cells migrate to the site of infection to exert their functions. Majority of the effector cells die while a small population of antigen-specific T cells persists as memory cells in distinct anatomical locations. The persistence and location of memory cells in lymphoid and non-lymphoid tissues is critical to protect the host from re-infection. The localization of memory T cells is carefully regulated by several factors including the highly organized secondary lymphoid structure, the cellular expression of chemokine receptors and compartmentalized secretion of their cognate ligands. This balance between the anatomy and the ordered expression of cell surface and soluble proteins regulates the subtle choreography of T cell migration. In recent years, our understanding of cellular dynamics of T cells has been advanced by the development of new imaging techniques allowing *in situ* visualization of T cell responses. Here, we review the past and more recent studies that have utilized sophisticated imaging technologies to investigate the migration dynamics of naïve, effector, and memory T cells.

## Imaging Technology

**Figure 1:**
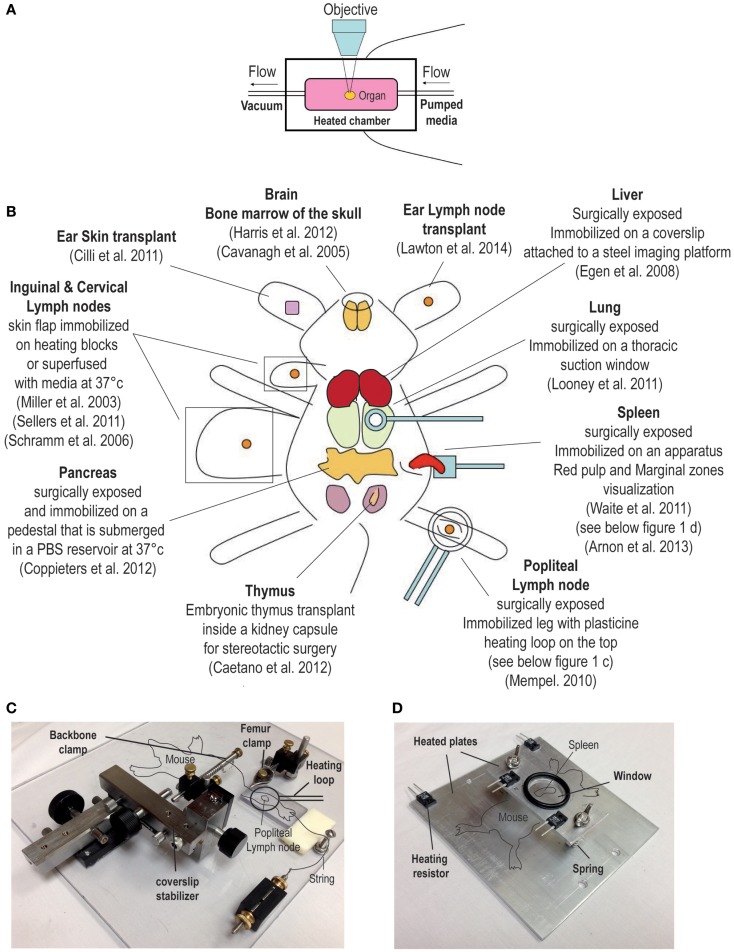
**Overview of surgical techniques for studying T cell dynamics**. **(A)** Explant system, the organ is kept in a heated chamber. Constant flow of warm media (bubble with 95% oxygen and 5% carbon dioxide) is maintained by a peristaltic pump. **(B)** Examples of intravital imaging methods previously used to observed T cell dynamic *in vivo*. **(C,D)** Examples of custom built stages used to immobilize the mouse for intravital imaging. **(C)** This stage has been used to image the popliteal lymph node; for a detailed description of this method, please refer to a publication by Murooka and Mempel (4). **(D)** The second stage is designed for imaging the spleen (5).

The ability to image the dynamics of T cell immune responses *in situ* has undergone significant advances over the past decade. For over a century, bright field transillumination or epifluoresecence microscopy was the only technology utilized to image excised organ sections or to visualize cellular processes *in vivo*. These techniques were useful for visualizing leukocyte interactions with the endothelium (1–3). The introduction of immunohistochemistry and immunofluorescence coupled with the use of monoclonal antibodies introduced specificity to the staining of lymphocytes. More recently, the advent of integrated fluorescent probes (e.g., CFSE) or natural fluorescent proteins (e.g., green fluorescent protein) permitted investigators to tag specific cell populations *in vivo*. These fluorescently labeled cells could now be tracked in real-time by directly imaging organs in explant preparations or directly intravitally in live animals. An overview of the techniques used for dynamic imaging of T cells is shown in Figure 1.

A significant technological advance was achieved with the laser scanning confocal microscope (LSCM). This type of microscope uses a lens to focus the laser excitation light on the specimen and the emitted light from the focal plane is refocused trough the same lens to the center of an open detector aperture (pinhole). This innovation obstructs the light coming from above and below the focal plane and thus increases the resolution. Sharp optical sectioning through a specimen at different depths can be performed to produce a 3 dimensional reconstruction of the sample. However, single photon confocal microcopy does not allow imaging at great depth (>100 μm) due to light scattering, photobleaching of stained tissue that is outside of the focal plane, and slow speed of data acquisition. Thus, it is very suitable for imaging thin tissues sections. Real-time dynamic imaging using LSCM is limited to the surface of the organ and for shorter periods of time. However, recent modifications to the standard single photon confocal microscope such as the addition of a microlens high speed spinning disk prevents cell damage and allows for rapid acquisition of imaging data of very large surfaces (approximately 870 μm × 660μm) (6). Thus, if deep tissue imaging is not required, the spinning disk confocal microscope can be very effective for performing dynamic imaging of large areas of various tissues. Several groups have recently used this technology for *in vivo* imaging, since it allows superior resolution (7). In a recent study, Cockburn and colleagues described the antigen-specific CD8+ T cell mediated killing of liver stage malaria parasites using a high speed spinning disk confocal microscope (7). In this case, even a superficial penetration of the laser beam was sufficient to observe the morphology of the liver parenchyma.

Compared to conventional lower wavelength and single photon excitation, the use of near-infrared two-photon (2P) excitation permits imaging of tissues at substantially greater depth (>300 μm). Moreover, the fact that the excitation of fluorescent proteins is confined to the focal plane significantly minimizes the problem of photobleaching. Consequently, by using 2P microscopy it is now possible to visualize the dynamics of immune cells in real-time, and at greater depths in intact explanted tissues or in live animals without causing overt cellular damage (8). Readily available tissues like the skin and the associated draining lymph nodes (dLN) were among the first tissues that were imaged intravitally using elegant surgical techniques (Figure 1). More recently, 2P microscopes have been modified and used to image several non-lymphoid tissues such as the lung, the intestines, the brain, and the liver (Figure 1) (9–12). 2P microscopy can also be used to visualize non-centrosymmetric structures such as collagen fibers (13). Non-linear optical effect called second harmonic generation (SHG) can be used to image collagen bundles in muscle and in bone tissues. When using a 2P laser, the emission of the SHG signal is exactly half of the excitation wavelength and can be very useful for providing structural reference of most tissues being imaged *in vivo* (14).

T cells are constantly moving inside and between organs, they are among the most motile cells in the body (an average of 10 μm/min, with peak velocity as high as 25 μm/min in the LN) (15). For this reason, the use of 2P microscopy has been a critical tool that has significantly increased our understanding of the dynamics of T cell responses *in vivo* (8, 16, 17). The disadvantages of this technique are the cost, and the limitation of the available fluorescent reporter mice or fluorescent probes.

## Surgical Techniques to Study T Cell Dynamics *in situ*

Among the first techniques used for observing T cell dynamics *in situ* was the organ explant system (Figure 1A) (18). It consists of a heated imaging chamber in which an organ such as a LN is immobilized and the chamber is then perfused with heated oxygenated media. This method offers greater stability and is suitable for imaging number of lymphoid and non-lymphoid tissues (11, 15, 19–21). However, excised organs that are submerged in a media filled chamber lack major vascular innervations such lymphatics and blood vessels. Moreover, chemokine production and distribution within the organ may be completely disrupted, and thus, the milieu in the excise organ may not reflect the tissue environment that exists *in vivo* in live animals. Moreover, in certain situations the dynamics of T cell behavior depends on the forces exerted by the fluid circulation. The best example is leukocytes extravasation from blood circulation into the underlying tissues where shear forces play an important role (22). Thus, intravital microscopic techniques to image myriad of different organs have been developed by several investigators (an overview is shown in Figure 1B) (23–25). As noted earlier, any studies that investigate the role of chemokines in regulating T cell migration will benefit from intravital microcopy since chemokine and the cytokine milieu can change drastically after an organ is removed. However, intravital microscopy involves complicated surgical techniques that can be invasive and cause vascular damage. As a result, several controls have to be performed and the experiments have to be repeated many times. In addition, other issues associated with intravital imaging must be considered; for example, the protracted anesthesia induced unconsciousness can decrease the heart rate impacting normal levels of blood circulation and ambient body temperature (26). However, it is possible to detect vascular leakage within the tissue being imaged by the systemic injection of fluorescent quantum dots. Local body temperature can be measured by the use of portable thermometers and constant temperature can be maintained by the use of a heated stage (Figures 1C,D) (5, 24). Certain organs (i.e., thymus) within their normal bodily context simply cannot be accessed for intravital imaging. For this reason, several groups have developed transplantation methods to provide better access of the organ for imaging (27, 28). For instance, a recent report described a thymic transplant on the kidney capsule of a nude mouse (that lacked an endogenous thymus) enabling better access of the organ for intravital imaging (27). Another group designed a thoracic suction window that stabilized the lung tissue in a live mouse without overtly disrupting the function (9). Although these surgical approaches for *in situ* imaging of lymphocytes are technically demanding, they have greatly facilitated our ability to observe T cell behavior directly *in vivo*.

## Visualizing Antigen-Specific T Cells *in situ*

The interaction of the T cell receptor (TCR) expressed on CD4+ and CD8+ T cells with a cognate peptide bound to a major histocompatibility complex (MHC) on an antigen presenting cell (APC) is essential to initiate the signaling cascade that eventually leads to T cell activation. Since, at steady state the precursor frequency of a naïve antigen-specific T cell population for a given epitope is extremely low (29, 30), adoptive transfer of labeled or congenically mismatched antigen-specific T cells isolated from TCR transgenic mice into a wild-type host has been a very useful tool for visualizing T cell dynamics *in vivo* (31–33). However, studies using adoptive transfer of TCR transgenic T cells are associated with certain caveats. Transfer of large numbers of naïve TCR transgenic T cells do not reflect the physiological precursor frequency, and will likely fail to mimic normal T cell responses (34). In addition, transferred TCR transgenic T cells express TCRs that exhibit identical affinity or avidity for a particular antigen, and thus, may not reflect a more physiological polyclonal endogenous T cell response to a pathogen (35). Nevertheless, in the absence of alternative technologies, in order to visualize the initial T cell activation (within minutes to hours after immunization), transfer of large numbers of TCR transgenic T cells is required.

Another major advance in detecting antigen-specific T cells was the development of MHC-multimers (36). An MHC monomer binds poorly to a specific TCR, while a multimeric MHC molecule binds stably to TCRs expressed on T cells and thus can be used effectively to stain antigen-specific TCRs allowing the detection of endogenous antigen-specific T cells. MHC class I tetramers have largely been used in flow-cytometric analysis, however, our group and others have effectively used *in situ* MHC-I tetramer staining for static imaging studies (37–39). Using this technique, we have previously visualized the anatomical program followed by endogenous antigen-specific CD8 T cells during a primary and a memory immune response against *Listeria monocytogenes* (LM) in the spleen (38). For a list of seminal publications that have contributed to the advancement of T cell imaging *in situ* please refer to Table 1.

**Table 1:** **Advances in *in situ* T cell imaging**.

Category	Year	Advancement	Imaging technique	Method	Organ imaged	Reference
Imaging T-cells – the beginning	1839	First *in vivo* imaging	Bright field	Intravital	N/A	(1)
	1994	First TCR transgenic adoptive transfer	Bright field	Immuno- histochemistry	Brachial LN	(31)
	1996	Intravital video microscopy	Bright field/EF	Intravital	Inguinal LN	(3)
Naïve T-cells in lymphoid tissues	2002	Real-time imaging of thymocytes positive selection	2P	Thymic organ culture	Thymus	(46)
	2002	T/B cell random walk in the lymph node cortex	2P	Explant	Inguinal LN	(15)
	2003	Intravital imaging of T cell trafficking	2P	Intravital	Inguinal LN	(109)
	2006	Lymphocyte migration along FRC in the LN cortex	2P	Fixative perfusion	Popliteal LN	(50)
			EM	IF	
			LSCM	
	2009	T cell egress from the LN	2P	Explant	Inguinal LN	(56)
T-cell priming/effector T cells	2002	Static imaging APC–T cell priming	LSCM	IF	Popliteal LN	(32)
	2002	Dynamic imaging of APC–T cell interaction at the LN surface	LSCM	Explant	Popliteal LN	(110)
	2003	Dynamic APC–CD8 T cell interactions in the LN cortex	2P	Explant	Inguinal LN	(58)
	2004	Intravital imaging of APC–CD8 T cell interaction in the LN cortex	2P	Intravital	Popliteal LN	(33)
	2005	Dynamic imaging of T/B cell conjugates in the LN	2P	Explant	Inguinal LN	(111)
	2006	Chemokine-driven non-random cell–cell interactions, initiating priming	2P	Intravital	Popliteal LN	(62)
	2007	Endogenous CD8 T cell activation following infection *in situ*	LSCM	Whole-mount, MHC-I tetramer staining	Spleen	(38)
	2008	Dynamic imaging of APC–CD8 T cell interactions in the splenic whit pulp	2P	Vibratome-cut explant	Spleen	(21)
	2011	Intravital APC–CD8 T cell interaction after LM infection	LSCM	Intravital	Spleen	(72)
	2011	Chemokine-induced optimization of CD8 T cell–APC interaction	2P	Intravital	Inguinal LN	(65)
	2012	Intranodal migration control T helper 1 differentiation	2P	Intravital	Popliteal LN	(61)
	2013	T cell–T cell interaction drive protective CD8 T differentiation	2P	Intravital	Popliteal LN	(66)
Naïve and effector T cells in non-lymphoid tissues	2008	Effector T cell dynamics in mycobacterial granulomas	2P	Intravital	Liver	(12)
	2011	Naïve and effector T cell dynamics in intact lung	2P	Intravital	Lung	(9)
	2011	Dynamics of primed CD8 T cell response during allograft rejection	2P	Intravital	Skin transplant	(100)
	2012	Effector T cell migration in *T. gondii* infected brain	2P	Explant and intravital	Brain	(11)
Memory T cells	2001	Generation of memory T cells in whole mouse body	EF	Sections	Whole body	(112)
	2011	Dynamic imaging of memory CD4 and CD8 T cells in skin	2P	Intravital	Skin	(96)
	2012	Chemokine-guided response of Central Memory T cells (T_CM_) to antigenic challenge	LSCM	Tissue sections and intravital	Popliteal LN	(71)
			2P	
	2013	Chemokine-dependent peripheral localization of CD8 memory T cells in lymph node	LSCM	Tissue sections and intravital	Popliteal LN	(64)
			2P			

*The table lists important advances in imaging of T cells in lymphoid and non-lymphoid tissues. The references represent to the best of our knowledge, the initial seminal research article published on the particular topic and the technique listed. 2P, two-photon microscopy; APC, antigen presenting cell; EF, epifluorescence; LN, lymph node; LSCM, laser scanning confocal microscopy; IF, immunofluorescence; FRC, fibroblastic reticular cell network*.

## Analysis and Data Interpretation

Observing the orchestrated movement of immune cells within intact organs without disrupting intricate organ structure is a powerful benefit of using 2P microscopy. However, imaging techniques described above offer a full spectrum of parameters that have to be effectively analyzed to obtain physiologically relevant and reliable data. Real-time imaging requires the acquisition of four dimensional data (*x*, *y*, *z*, *t*; time), which can be used effectively to quantify cellular dynamics such as cell–cell interactions, cellular velocity, cellular contact time, chemotactic and shape index, and much more. For a more thorough review of this topic readers should refer to previously published reviews (8, 40). However, it is noteworthy that two groups recently combined flow cytometry and *in situ* imaging (41, 42) to develop a novel way to analyze imaging data. The first group published the “histo-cytometry” method, which was applied to investigate dendritic cell (DC) subset localization in the LN. DCs represent a highly heterogeneous population of cells and thus it is necessary to stain for at least five markers to identify several specific subsets. Using this novel method they were able to gate on a specific DC subset and simultaneously analyze the localization of the particular population directly within the LN section (42). The second group, Moreau et al. developed the so-called “dynamic *in situ* cytometry” (DISC), by combining 2P imaging with direct *in vivo* staining by injecting Fab fragments of antibodies against cell surface molecules of interest. By converting files that represent imaging data into the regular FCS format, data were easily analyzed using a flow-cytometric software. As a consequence, cell phenotype was effectively linked to *in situ* cell behavior (41). In addition, another elegant method termed, the “intravital dynamics-immunosignal correlative microscopy” linked dynamic behavior of T cells with static antibody stained imaging. Chodaczek and colleagues fixed the whole tissue immediately after dynamic imaging and proceeded to stain the fixed tissue with antibodies to specific proteins. By using tissue landmarks they were able to realign the T cell movements with static immunofluorescent images, and thus, single cell dynamic behavior was effectively linked to the location of TCRs and signaling molecules *in situ* (43).

## Visualization of T Cell Responses in Lymphoid Organs

### Thymus

Early histological studies using fixed thymic sections revealed the geographical location of developing thymocytes *in situ* (44). It was demonstrated that double-negative (DN) thymocytes spent an average period of 14 days before becoming double-positive (DP) cells at the corticomedullary junction. In the next 3–5 days these DP cells were shown to migrate to the cortex where they interacted with the cortical thymic epithelial cells (cTEC) and underwent positive selection and matured into single-positive (SP) thymocytes. The final process of negative selection occurred in the thymic medulla where SP thymocytes interacted with the medullary thymic epithelial cells (mTECs), before exiting to the periphery (45).

Recent studies using 2P dynamic imaging have significantly increased our understanding of the T cell developmental process by defining thymocyte–APC interactions and trafficking patterns within the thymus. The first real-time imaging study utilized reaggregate thymic organ cultures to characterize the dynamics of thymocyte behavior during the process of positive selection (46). The same group further extended their findings by using thymic explants and 2P microscopy (19). They showed that T cells located in the thymic cortex exhibited the same stochastic migration pattern previously observed in the LN cortex (15), but the cells moved at a relatively low speed of 3–8 μm/min. However, after undergoing positive selection, T cells exhibited significantly higher motility of 10–25 μm/min as they migrated toward the medulla. Since the thymic medulla is located at greater depth, dynamic 2P imaging of this region required the use of vibratome-cut thymic explants (47). Intriguingly, SP thymocytes undergoing negative selection were confined to a specific area of the thymic medullary region and exhibited an average velocity of 10 μm/min. By using a mouse model that expressed the ovalbumin (OVA) antigen in mTECs, Le Borgne et al. visualized the process of negative selection of OVA specific TCR transgenic CD8 T cells (OTI) *in situ*, and showed that negatively selected T cells surprisingly continued to stay motile but were confined to a restricted area of the medulla. This observation implied that SP thymocytes needed continuous cellular interactions and integrations of signals before undergoing apoptosis (47).

### Lymph node

The emerging data indicate that mounting a protective immune response against pathogens or tumors is critically dependent on the orchestrated movement of cells within lymphoid organs. The lymph node structure is one of the underlying regulators of immune responses against mucosal infections or following vaccination by promoting interactions between different cell types. Thus, understanding the dynamics of T cell behavior *in situ* within the LN is essential. Skin draining LN can be accessed for intravital imaging, therefore, several previous studies have reported intranodal T cell behavior in several different contexts (48).

#### Naïve T cell trafficking

T cell trafficking patterns even under steady state conditions is a highly regulated process and several recent elegant studies have helped illuminate the processes that control this complex behavior of T cells *in situ*. Naïve T cells access the LN via the blood and enter the LN cortex through the high endothelial venules (HEV). Once in the cortex, T cells scan the DC networks (49) for antigen as they follow the fibroblastic reticular network within the LN (50). Factors that govern this migration pattern are not completely understood (48); however, G-protein coupled receptors (GPCRs) are very important in regulating this process. By abrogating global GPCRs signaling with pertussis toxin (PTX) treatment, Cyster and colleagues showed that PTX treated T cells showed a 50% reduction in median velocity, and a 90% decrease in mean motility coefficient in the LNs when compared to untreated T cells (51). Among the different GPCR, CCR7 was shown to be important for the localization of T cells in the paracortex. Indeed, modification of the CCR7 ligand (CCL19 and CCL21) distribution by subcutaneous injection distracted the lymphocytes from the T cell zone (52). The absence of the CCR7 signaling on naïve T cells significantly reduced the intranodal normal T cell velocity. However, this deficiency did not introduce any directional biases (52, 53), and thus, the “random walk” behavior exhibited by T cells was unchanged. Although both ligands for CCR7, CCL21, and CCL19 are produced by FRCs (54) only surface bound CCL21 is require for the random T cell motility (55) within the LN. These observations suggest that T cells follow a haptotactic (immobilized ligand) instead of a chemotactic (soluble ligand) gradient.

Egress of naïve T cells is also regulated by GPCRs. Upon LN entry, naïve T cells spend on average 6–12 h in the LN cortex, before using the cortical lymphatic sinuses to exit the LNs. Cyster and colleagues elegantly visualized this process, and showed that naïve T cells first probed the cortical lymphatic sinuses, and subsequently entered the lymphatic vessels by a sphingosine-1-phosphate receptor-1 (S1PR1) dependent mechanism (56). The S1PR1 ligand shingosine-1-phosphate (S1P) is present at high concentrations in the blood and in the lymph, but virtually absent in the tissues due to the degradation by the enzyme S1P lyase (57). S1PR1 is rapidly desensitized after S1P ligation, thus newly arrived T cells in the LN cortex fail to express the receptor on their surface. Once S1PR1 is recycled back to the surface, T cells are able to respond to the S1P gradient and return back to the circulation following exit from the LNs.

#### Naïve T cell priming

Visualizing T cell activation *in situ* has considerably enhanced our understanding of the mechanisms that regulate T cell and APC interactions *in vivo*. Three major experimental models have been utilized to image T cell priming; subcutaneous antigen delivery coupled with adjuvant, transfer of antigen pulsed DCs or direct infection of animals with pathogens.

Early 2P microscopy studies using antigen pulsed DCs to prime T cells revealed the dynamics of T cells–DC interactions *in situ* (33, 58) during antigen presentation. Antigen-specific T cells formed protracted interactions with DCs that lasted not minutes but hours (33, 58). Mempel et al. described a more complex process that characterized the dynamics of T cell activation. They demonstrated that T cell priming occurred in distinct phases where initial repeated brief encounters with DCs was followed by long-lived stable DC–T cell conjugates that in some instances lasted for more than 20 h in the LNs (33). The stability of these interactions depends on the antigen dose and TCR–MHC affinity (59, 60). Chemokines are also important for promoting T cell–APC interactions. CD4 T cell that are deficient in CXCR3 display fewer and shorter interactions with DCs that expressed the cognate ligand CXCL10, which resulted in poor Th1 differentiation as well as misplaced intranodal migration of primed CD4 T cells (61). In addition, collaboration between lymphocyte subsets was shown to facilitate antigen recognition of rare antigen presenting DCs at early stages of an immune response in the LN, resulting in non-random cell–cell interactions (62). Early during an immune response, the interaction of CD4 T cells with antigen bearing DCs resulted in the production of chemokines CCL3 and CCL4. This in turn lead to the recruitment of CCR5 expressing CD8 T cells to these rare sites of antigen depot in the LN, allowing for optimized T cell priming and memory cell generation (62).

Thus, the emerging data suggest that the “dwell time” (the length of initial T cell–DC conjugate formation) and the subsequent T cell motility and migration within defined compartments of secondary lymphoid organs are important parameters directing optimal T cell activation *in vivo* and these parameters are sensitive *in situ* indicators of antigen recognition.

In limited number of cases T cell dynamics in secondary lymphoid organs has been investigated following an infection. Although imaging studies using simple antigens have improved our knowledge regarding the mechanics of T cell activation, observing T cell responses to live replicating pathogens adds new layers of complexity. Since naïve T cells are largely located in the LN cortex where they continuously scan DCs, it was tempting to assume that following infection, T cell priming would occur deep in the LN cortex. However, *in vivo* visualization of early T cell–APC interactions immediately following viral infections revealed that T cell priming in fact occurred near the cortical ridge or at the interfollicular region of the dLN (32, 63–65). Subcutaneous infection with vaccinia virus or vesicular stomatitis virus (VSV) resulted in the infection of macrophages and DCs present in the LN subcapsular sinus (SCS), however, only DCs that expressed virus encoded protein appeared to present antigen directly to transferred TCR transgenic CD8 T cells (32, 63, 65). Interestingly, acquisition of effector functions and proper differentiation of primed CD8 T cells during the later phases of the T cell priming process depends on T cell–T cell interactions rather than just the early DC–T cell interactions (66). Integrin (CD11a) mediated T cell homotypic interactions was shown to be essential for the ability of antigen-specific CD8 T cells to secrete interferon-γ and subsequently differentiate into memory T cells following infection.

Following infection, effector T cells proliferate briefly in the LNs before exiting via the efferent lymphatics into the circulation. Most studies have focused on early events using TCR transgenic adoptive transfer methods but the subsequent intranodal migration of newly primed T cells, or the mechanisms that drive the egress kinetics from the LN following a localized infection have not been investigated adequately. However, a recent study used antigen pulsed DCs to immunize mice and demonstrated that stromal cells and DCs in the interfollicular area express CXCL9 and CXCL10, respectively, and attract newly primed CXCR3 expressing CD4 T cells to the interfollicular and medullary areas of the LN (61). In this case, cell–cell interactions in the periphery of the LN were important for the proper intranodal T cell positioning as well as adequate Th1 cell differentiation.

Thus, these observations clearly demonstrate the complexity of the T cell activation process that requires several types of dynamic interactions between immune as well as stromal cells. Clarification of these elaborate processes has only been made possible by the use of *in situ* imaging methods.

#### Central memory T cells

Following the resolution of an acute infection, a heterogeneous population of memory T cells are generated that exhibit differential tissue tropism (67–70). Central memory T cells (T_CM_) that express the lymphoid homing receptors CCR7 and CD62L preferentially localize to the secondary lymphoid organs, while effector memory T cells (T_EM_) that fail to express lymph node homing receptors migrate to non-lymphoid tissues where they may upregulate CD69 and CD103 and form a stable resident memory T cell population. Central memory CD4+ and CD8+ T cells (64, 71) are largely located in the periphery of the LNs in close proximity to the lymphatic sinuses where a potential re-infection may occur. This peripheralization was shown to be dependent on the expression of the chemokine receptor CXCR3. Thus, this “pre-positioning” of memory T cells allowed for a more rapid response to a challenge infection.

### Spleen

Following a blood borne infection, the spleen plays an essential role in the initiation of an anti-microbial immune response. A systemic infection with the intracellular Gram-positive bacteria LM is a widely used model to study the immune responses in this important lymphoid organ. Contrary to the LN, the spleen is more difficult to access for intravital imaging. Moreover, light scattering and absorption by red blood cells makes multi-photon microscopy of the spleen challenging. Additionally, the splenic white pulp, which plays host to T and B cells, is too deep to be imaged directly by 2P microscopy. However, using a standard confocal microscope the splenic red pulp was imaged intravitally following LM infection (72). During early time points after infection, DCs in the splenic red pulp established static LM depots, which were subsequently swarmed by neutrophils and monocytes, as well as CD8+ T cells (72). At later times after infection, infected DCs migrate to the splenic white pulp where they form stable interactions with CD8+ T cells, resulting in T cell activation (21, 73).

Using *in situ* tetramer staining and static whole-mount confocal microscopy with thick spleen sections, we have previously mapped the entire anatomical program followed by *endogenous* antigen-specific CD8+ T cells in the spleen after a primary and secondary LM infection (38). At day 3 after infection tetramer positive (tet+) cells were readily detected in small clusters at the T/B cell border and in the splenic marginal zone in close contact with DCs. After a brief period of expansion in the T cell zones, by 6 days after infection, virtually all antigen-specific effector CD8+ T cells had exited the white pulp using the bridging channels (lymphatic vessels are absent in the spleen). Similar to what was observed in the LN (64, 71), we found that early memory T cells were preferentially localized to the periphery of the splenic T cell zones and surprisingly even in the B cell follicles. Interestingly, another study showed that memory CD8 T cells beyond 55 days post LCMV infection were primarily located in splenic T cells zones (74), suggesting that as memory T cells mature their anatomical localization may change. This process will likely depend on the differential expression of homing and chemokine receptors that are involved in memory cell localization and migration.

## Imaging T Cell Responses in Non-Lymphoid Organs

Following an infection, effector T cells, apart from proliferating in lymphoid organs, also migrate to peripheral inflamed tissues via the vasculature. Several adhesion molecules and chemokines are involved in the migration and entry of effector T cells into peripheral tissues. Receptors like E-selectin and P-selectin expressed on endothelial cells within tissues and molecules like CD44, P-selectin glycoprotein ligand 1 (PSGL1) and α4β1 integrin on antigen-challenged T cells enable non-selective localization of T cells to various peripheral organs (75). However, tissue-specific chemokines and receptors expressed on T cells specifically direct the homing of activated T cells to a particular non-lymphoid organ such as the skin, brain, or the gut (76). E-selectin ligands and the chemokine receptors CCR4 and CCR10 direct T cell homing specifically to the skin, while CXCL10 and CXCR3 regulate recruitment to the brain (11). At mucosal sites such as the intestinal mucosa, DCs in the Peyer’s patches or mesenteric LNs imprint T cells to specifically migrate to the intestinal tissue (77). These mucosal activated T cells upregulate CCR9 and α4β7, which allow the T cells to home to the gut mucosa. α4β7 binds to the mucosal addressin cell adhesion molecule-1 (MADCAM-1), which is expressed heavily on vascular endothelial cells in the intestinal tissue (78), while CCL25 (CCR9 ligand) is produced by the intestinal epithelial cells (79).

### Effector T cells

Once effector T cells enter peripheral tissues, they search for their cognate antigen, which is presented in context with MHC molecules by APCs or tissue stromal cells. Advanced imaging techniques like intravital imaging and multi-photon microscopy have attempted to shed light on the behavior of T cells navigating peripheral tissues (11, 16, 80). 2P imaging in the brain showed CD8+ T cells undergoing a non-random, directed mode of migration (11). The authors speculated that this unusual pattern of migration called a “Levy walk” enabled T cells to find rare APCs faster. In this particular study, the observed Levy walk helped T cells control *Toxoplasma gondii* infection in the brains of infected mice. Analysis of cell motility in non-lymphoid tissues revealed that T cells move at an average velocity that is significantly lower than their counterparts in the LNs. Intravital imaging in the lung was used to image naïve and activated T cells. Naïve T cells maintained an average track speed of around 2.5 μm/s, whereas activated T cells moved at a substantially lower speed of 0.4 μm/s (9). In the liver, granuloma formation following infection with *Mycobacterium bovis* (BCG) has been used as a model system to image the dynamics of innate and adaptive immune cells (12, 81). The study revealed fast but restricted movement of CD4+ T cells inside granulomas (12). The same group later demonstrated that antigen presentation within granulomas determined T cell motility and cytokine production (81), which in turn influenced pathogen clearance. Thus, it is clear that tissue microenvironment likely influences T cell motility. There are several factors that can influence the differential dynamic behavior of T cells in non-lymphoid tissues. These factors include but not limited to: (i) nature of the replicating pathogen with regards to cellular tropism and the inflammatory milieu the pathogen may induce; (ii) the production of chemokines and their receptors expressed on T cells (62, 82); and (iii) the tissue resident cells including stromal or APCs that T cells may interact with (83). Determining the role each of these factors play in regulating T cell dynamics in non-lymphoid tissues will be essential for closing the large gaps that exist in our knowledge regarding T cell behavior in non-lymphoid tissues.

Non-T cell populations like neutrophils and DCs in the lung and DCs in the gut lumen have also been studied using 2P imaging (9, 84–88). However, as the focus here is on T cells, a detailed review of non-T cell populations is beyond the scope of this article.

### Memory T cells

After effector T cells perform their function of antigen recognition, interaction, and clearance, a subpopulation of T cells persist as memory cells. A hallmark of protective immunity is that these cells help mount a rapid immune response following a secondary infection. As noted earlier, memory cells can be divided into T_CM_ and T_EM_ cells. T_CM_ re-circulate predominantly between secondary lymphoid organs and the blood, whereas T_EM_ re-circulate through peripheral non-lymphoid tissues (68). A third category of memory cells are called resident memory cells (T_RM_) that remain in peripheral tissues and fail to re-circulate (89–93). Understanding the receptors, chemokines, and other factors that influence formation of a T_RM_ population in non-lymphoid tissues is an area of active research. Intra-epithelial lymphocytes (IELs), the T_RM_ cells in the gut epithelium, downregulate expression of homing receptors α4β7 and CCR9 and upregulate CD69 and CD103 to establish residence (89, 94). Decrease in the expression of KLF2, the transcriptional activator of the gene that codes for S1PR1 was recently shown to be important for establishment of resident memory CD8+ T cells (95) in non-lymphoid tissues. Since S1PR1 plays an important role in naïve T cell egress from lymph nodes (56), transcriptional downregulation of this gene for the generation of resident memory population is an important and interesting observation. 2P microscopy has helped address previously unappreciated questions about the nuances of memory T cell dynamics in non-lymphoid tissues following infection. After HSV infection in the skin, memory CD4+ and CD8+ T cell populations adopt different rates of migration based on tissue localization. CD8+ memory T cells remain in the epidermis close to the initial site of infection and migrate at a slower rate (2–3 μm/min) than dermis-localized CD4+ T memory cells (5–6 μm/min) (96). Interestingly, CD4+ T cells could re-enter the blood circulation, whereas CD8+ cells did not, suggesting that CD4 and CD8 T cells are regulated differently with regards to tissue residence. The mechanisms responsible for these differences are not known. As a follow-up, recent published work using multi-photon imaging and computer modeling showed that the slower velocities of CD8+ T_RM_ cells in the epidermis enabled these cells to remain on site throughout the life of the mouse (97). The observed cell velocities obtained *in vivo* over 2–3 h were used to model migration patterns over longer periods of time. The mathematical modeling showed that percentages of CD8+ T cells within a 5-μm region of skin at day 100 (49.8%) and day 365 (49.4%) after infection were not significantly different from day 0 (50%). Furthermore, two-photon imaging also demonstrated that the CD8+ T_RM_ population interacted with and were influenced by other cell populations like Langerhans cells (LCs) and dendritic epidermal γδ T cells in the epidermis.

### Autoimmunity, tissue rejection, and tumor immunity

The immune system can sometimes backfire, leading to autoimmunity and medical complications like graft rejection. Using mouse models and novel imaging tools, efforts are being made to understand these processes better. In a diabetic mouse model, 2P microscopic study showed that cytotoxic T lymphocytes (CTLs) underwent random walks in the exocrine tissues of the pancreas. On encountering β-cells in the pancreatic islets, the CTL motility slowed and led to the eventual death of β-cells, causing diabetes (98). In experimental autoimmune encephalomyelitis (EAE), a mouse model for multiple sclerosis, a molecular sensor that combines fluorescent nuclear factor of activated T cells (NFAT) with the histone protein H2B was used to detect intravital T cell activation (99). In activated T cells, NFAT is dephosphorylated and translocates into the nucleus. Tagging NFAT with fluorescent histone 2B enables simultaneously tracking NFAT nuclear translocation and cell mitosis, a powerful tool to follow activation of individual T cells *in vivo* following CNS invasion during development of EAE. Initially, T cells that traversed the leptomeningeal blood vessels in the CNS were activated following transient contacts with resident macrophages. During disease progression, activated T cells spread to the CNS parenchyma. In another study, the step-wise host tissue destruction was studied in a mouse model of allograft rejection, where ear skin grafts were imaged using intravital 2P microscopy (100). Donor dermal DCs were destroyed within 3–5 days after transplant. Rapid infiltration of host CD11b+ cells, initially neutrophils replaced by monocytes, could act as APCs that transported antigen from graft to the dLN, thereby activating CD8+ T cells. The above-mentioned two studies, therefore, demonstrated how intravital imaging and multi-photon microscopy could provide crucial temporal information on important processes like disease progression and graft rejection.

T cell homing and infiltration of tumors play an important role in improved cancer prognosis. Receptors like CXCR3, CCR5, CCR4, and adhesion molecules like intercellular adhesion molecule-1 (ICAM-1) have been implicated in T cell infiltration to various types of tumors (101, 102). Cytotoxic T lymphocytes (CTLs) infiltrate tumors and mediate tumor cell destruction. Multi-photon microscopy has been used effectively to image the dynamics of CTLs within solid tumors. In a mouse thymoma model, E.G7-OVA tumor cells (modified to express CD8 T cell antigen OVA) were injected into mice subcutaneously (s.c), and 2P microscopy was utilized to assess OVA specific CD8 T cells (OTI) dynamics during early and late phases of tumor rejection. OTI arrest coefficient was higher, while the mean velocities were decreased in the OVA-expressing tumors during early stage of rejection, when compared to non-OVA bearing tumors, suggesting antigen-specific recognition by CD8 T cells was necessary for T cell arrest and the eventual destruction of tumor cells. (103). To better understand if CD8 T cells directly mediate tumor cell lysis, a combination of 2P microscopy and a fluorescence resonance energy transfer (FRET) based caspase-3 activity reporter system was used to track apoptosis of tumor cells following adoptive transfer of activated CD8 T cells (104). Indeed, this study revealed that CD8 T cells directly kill individual tumor cells, however, the time required for tumor cell lysis was unusually lengthy (6 h on average), which may explain why T cell mediated tumor regression is often inefficient. Other immune cell types that target tumor antigens, like natural killer (NK) cells, have also been imaged using multi-photon microscopy and were shown to have very different dynamics as compared to CTLs (105).

Real-time *in situ* imaging of tumor tissue has also shed light on potential mechanisms that can restrict CTLs from carrying out their antitumor function within solid tumors. Myeloid-derived suppressor cells (MDSCs) are heterogeneous populations of APCs such as tumor dendritic cells (TuDCs) or tumor-associated macrophages (TAMs), which can subvert the antitumor activity of CTLs (106). 2P imaging of tumor tissue showed that following chemotherapy, tumor-infiltrating lymphocytes (TILs) made increased contacts with TuDCs but were trapped in TuDC-rich areas in the tumor parenchyma, restricting their infiltration deeper into tumor tissue (107). Furthermore, in a mouse model of adoptive T cell transfer, intratumoral regulatory T cells (Tregs) in an antigen-dependent manner, induced a functional anergic state in tumor-infiltrating CTLs, which resulted in poor tumor regression (108). Thus, using 2P microscopy to image solid tumors has been a valuable tool for gaining critical information regarding the dynamic behavior of TILs *in vivo*.

## Conclusion

Two-photon microscopy and intravital imaging have helped make significant strides in increasing our understanding of the spatial and temporal behavior of T cells and APCs *in situ* during an immune response and subsequent steps of memory cell generation. With lesser restraints on the type and thickness of peripheral live tissues that can be imaged in real-time, the possibilities are limitless to address further unanswered questions regarding the biology of the immune response to a tumor or following an infection, and thus the development of effective vaccines and therapeutics.

## Conflict of Interest Statement

The authors declare that the research was conducted in the absence of any commercial or financial relationships that could be construed as a potential conflict of interest.
